# The Impact of Parental Relationship Satisfaction on Infant Development: Results From the Population-Based Cohort Study DREAM

**DOI:** 10.3389/fpsyg.2021.667577

**Published:** 2021-08-06

**Authors:** Caroline Nicolaus, Victoria Kress, Marie Kopp, Susan Garthus-Niegel

**Affiliations:** ^1^Institute and Policlinic of Occupational and Social Medicine, Faculty of Medicine, Technische Universität Dresden, Dresden, Germany; ^2^Department of Medicine, Faculty of Medicine, Medical School Hamburg, Hamburg, Germany; ^3^Department of Child Health and Development, Norwegian Institute of Public Health, Oslo, Norway

**Keywords:** relationship satisfaction, couples, parent-infant relationship, infant development, longitudinal research, DREAM study

## Abstract

Extensive literature has shown that interparental conflicts and violence have detrimental effects on children's adjustment in childhood and adolescence. It is not equally well-understood how parental relationship satisfaction impacts infant communicational and personal-social development during the first year of life. This longitudinal study examines (a) the impact of maternal and paternal relationship satisfaction on infant development, (b) whether this prospective association is mediated by parent-infant relationship, and (c) a potential moderating effect of infant gender. Data were derived from the population-based cohort study “Dresden Study on Parenting, Work, and Mental Health” (DREAM) including 1,012 mothers and 676 fathers. Relationship satisfaction and parent-infant relationship were assessed eight weeks postpartum, infant communicational and personal-social development were measured 14 months postpartum. Multiple linear regression, mediation, and moderation analyses were conducted for mothers and fathers separately. It was shown that paternal relationship satisfaction is a significant predictor of infant personal-social development. This prospective association was partially mediated by father-infant relationship. When postnatal depression was included in the analysis, however, father-infant relationship was not a significant mediator. The association in fathers is neither reduced nor increased as a function of infant gender. No similar effects were found in the mothers' sample. Parental relationship satisfaction did not significantly predict infant communicational development in either mothers or fathers. The study findings highlight the importance of paternal relationship satisfaction, father-infant relationship, and postnatal depression for infant personal-social development.

## Introduction

The family context forms the central living environment for young children and is often seen as the most proximal system by which children are socialized (Bush and Peterson, 2013). Hence, besides other social, environmental, and biological factors, the family system is an essential influential factor for child development (e.g., Bronfenbrenner, 1979; Feldkötter et al., 2019). On the one hand, it offers a fundamental resource pool, on the other hand, it represents a potential origin of risk factors for child development (O'Brien, 2005; Garthus-Niegel et al., 2017; Junge et al., 2017; Polte et al., 2019; Thiel et al., 2020). The first years of life are a particularly formative time during which certain experiences can have a maximally positive or negative impact on further life (Montada, 2008). Therefore, the experiences children have in their early years are of enormous importance for further development and long-term mental health (Harold et al., 2016). The strength and adaptive functioning of the inter-parental relationship can have particularly profound implications for child well-being and development (Grych and Fincham, 2001; Goldberg and Carlson, 2014). It is widely acknowledged that this relationship represents a key element in determining the quality of family life (Erel and Burman, 1995; Goldberg and Carlson, 2014; Feldkötter et al., 2019).

Emerging research indeed demonstrates an association between parental relationship and child development. Available evidence on negative features of the parental relationship, such as conflicts and violence, indicates that higher levels of unresolved conflict and discord are associated with various child emotional and behavioral problems, insecure attachment to parents, social difficulties, less academic and educational attainment, and worse relationship stability in later life (Fishman and Meyers, 2000; Harold et al., 2016). In contrast, the literature concerning positive aspects of parental relationship, such as intimacy or constructive communication, is sparse (Dominick, 2018). Studies suggest that parents' supportiveness of one another, positive affect, and the ability to resolve conflict and communicate effectively are positively linked to better child behavioral outcomes (e.g., Goldberg and Carlson, 2014; Feldkötter et al., 2019). Furthermore, Cowan and Cowan (2005) showed that strengthening the partner relationship positively impacts both child adjustment and parent-child relationship.

In this context, mechanisms of action, explaining how and why relationship quality is related to child outcomes, have yet to be identified. Family system theory emphasizes that individuals cannot be understood in isolation from one another, but rather as a part of their family. Thus, families are conceptualized as a dynamic and integrated whole, in which individual family members are influenced by one another (Minuchin, 1988; O'Brien, 2005). Changes in the relationship between certain family members are believed to affect the development and growth of other individuals within the family. Hence, the manner in which parents communicate and relate to each other is important for their children's development, because it affects children both directly and indirectly (Goldberg and Carlson, 2014). In terms of direct effects, social learning theory proposes that children imitate the behavior and interactions of significant others, especially their parents (Bandura, 1978). Therefore, loving and constructive interaction between parents may produce similar behavioral styles in children. In addition, spillover theory can provide an additional explanation for the link between parental relationship quality and child outcomes. It postulates that emotions or behaviors generated in one family subsystem are transferred to another within the family system (Harold et al., 2016; Dominick, 2018). Therefore, parental relationship also affects children's development indirectly through its influence on parenting behaviors. Accordingly, parental relationship quality may be directly related to specific parenting behaviors, because difficulties, stress, or anger produced in the dyadic couple relationship may carry over into the parent-child relationship, which in turn may impact child development. Spillover theory has received strong empirical support (e.g., Krishnakumar and Buehler, 2000; Bradford and Barber, 2005; Harold et al., 2016). Feldkötter et al. (2019) found that the negative association between relationship satisfaction and child problem behavior was mediated by negative parental behavior and parental stress. Similarly, Cox et al. (1989) showed that mothers who experienced their partnership as close and open-minded also expressed more warmth and sensitivity toward their infant during playing interactions. Howard and Brooks-Gunn (2009) noted that a supportive relationship between parents is associated with positive parenting behaviors. In turn, positive and empathetic interactions between the reference person and the child are an essential prerequisite for adequate communicative and personal-social development (Papoušek, 2006; Mensah and Kuranchie, 2013).

The association between parental relationship quality and child development may vary depending on child gender. Although both girls and boys are affected by parental relationship quality, they may respond differently (Grych et al., 2003). Previous research suggests that boys are more likely to perceive conflicts between their parents as a threat to themselves, whereas girls are more likely to interpret them as a threat to family harmony (Grych et al., 2003; Harold et al., 2016). In addition, compared to boys, girls may hold themselves more accountable for interparental conflicts (Buchanan et al., 1991; El-Sheikh and Reiter, 1996). However, past research yielded ambiguous results. For example, Davies and Lindsay (2001) showed that parental conflict (i.e., avoidance-surrender and verbal aggression) had a greater impact on externalizing problems in boys and internalizing problems in girls. In contrast, other studies found no significant differences regarding the association between parental relationship and well-being of female versus male children (Fishman and Meyers, 2000; Goldberg and Carlson, 2014). And still other studies found that interparental disagreements had a greater impact on boys (Emery and O'Leary, 1982; Reid and Crisafulli, 1990). Resulting from these discrepant findings, the effect of child gender on the prospective association between parental relationship quality and developmental outcomes requires explication.

Preceding research provides theoretical and empirical support for the notion that parental relationship quality plays an important role in child development. Nevertheless, previous research on the consequences and correlates of parental relationship satisfaction was primarily focused on late childhood and adolescence, including children aged 10 years and older. Research pertaining to early childhood, including infants and toddlers, is sparse (Dominick, 2018). Because the first years of life have a considerable impact on further development (Bedi and Goddard, 2007), studies focusing on the effects of parental relationship satisfaction on their children's first year are needed. This study extends existing literature by taking into account this prospective association during infancy. Additionally, the present study addresses the relationship of *positive* interparental interactions, which has received relatively little prior empirical attention. Whereas a large number of studies primarily examined marital conflict behavior and consequences of separation (Dominick, 2018; Feldkötter et al., 2019), the present study broadens the view and considers not only dispute behavior, but also positive features of the couple relationship, such as endearment and community/communication. Both parents are considered in this investigation to create a more comprehensive picture of the prospective associations between parental relationship satisfaction, parent-infant relationship, infant gender, and infant development.

In light of the empirical and theoretical considerations stated above, the aim of this longitudinal study is to examine the prospective association between maternal and paternal relationship satisfaction at eight weeks postpartum and infant development at 14 months postpartum, controlling for a set of potential confounders. It is hypothesized that higher levels of maternal and paternal relationship satisfaction are prospectively and directly associated with better infant communicational and personal-social development. In addition, the study aims to investigate whether infant gender moderates the direct association between parental relationship satisfaction and infant communicational and personal-social development. Due to prior mixed findings in this area, no specific hypothesis is postulated. Further, it is assumed that for mothers and fathers, parent-infant relationship mediates the positive association between parental relationship satisfaction and infant communicational and personal-social development, i.e., higher levels of parental relationship satisfaction are related to better parent-infant relationship, which in turn is linked to positive infant developmental outcomes.

## Materials and Methods

### Design

Data were derived from the Dresden Study on Parenting, Work, and Mental Health (“**DR**esdner **S**tudie zu **E**lternschaft, **A**rbeit und **M**entaler Gesundheit,” **DREAM**). This prospective multi-method cohort study examines “the relationship between parental work participation, role distribution, stress factors, and their effects on perinatal outcomes and long-term family mental and somatic health […]” (Kress et al., 2019, p. 1). Expectant mothers and their partners were recruited during pregnancy, mostly at obstetrical clinics' information events and birth preparation courses in and around Dresden, Germany. DREAM is a longitudinal study consisting of six measurement points spanning over the course of pregnancy to 4.5 years postpartum. Various questionnaires are completed by (expectant) mothers and their partners at each time point.

The current investigation included data of the first three measurement points: T1: during pregnancy, T2: eight weeks after the anticipated birth date, and T3: 14 months postpartum. Further details regarding DREAM study design and measures can be found in the study protocol (Kress et al., 2019) and Supplementary Figure 1.

### Participants

The present study included data from women and men who were expecting one child, completed T1–T3, and indicated being in a relationship with the other biological parent. The retention process is presented in Supplementary Figure 2. Mothers and fathers could take part in the study alone or with their partner. At the time of data extraction (3rd of December 2020), *n* = 1,065 women and *n* = 695 men completed T3 in time. Because of the aforementioned inclusion criteria, *n* = 24 (2.3%) mothers and *n* = 12 (1.7%) fathers with twins or multiples were excluded, as well as *n* = 28 (2.7%) mothers and *n* = 6 (0.9%) fathers who were not in a relationship with the other biological parent. Likewise, *n* = 1 (0.0%) mother and *n* = 1 (0.1%) father were excluded because their infant was living separated from them. The final sample included *n* = 1,688 parents, consisting of *n* = 1,012 mothers and *n* = 676 fathers.

### Measures

*Parental relationship satisfaction* was measured via the validated German short version of the relationship questionnaire (“Kurzform des Partnerschaftsfragebogens,” PFB-K; Kliem et al., 2012) at T2, i.e., eight weeks after the anticipated birth date. The questionnaire consists of three subscales (endearment, dispute behavior, and community/communication) with three items, respectively. Each item was rated on a scale from 0 (*never/very rare)* to 3 *(very often)*, and the total score for the whole scale was obtained by summation of all items ranging from 0–27. Higher scores reflect higher levels of parental relationship satisfaction. In the current sample, internal consistency was high for both mothers (α = 0.80) and fathers (α = 0.78).

*Parent-infant relationship* was measured with the German version of the Postpartum Bonding Questionnaire (PBQ; Reck et al., 2006) at T2. The PBQ was designed to provide an early indication of disorders within parent-infant relationships (Brockington et al., 2001), comprising 25 items on four dimensions, namely *general factor, rejection and anger, anxiety about care*, and *risk of abuse*. Parents are asked to think of the most difficult time with their infant and to rate how frequently they experience each situation. Items are scored from 1 (*always*) to 6 (*never*), with the total score ranging from 0–125. In the current study, items were recoded so that higher values represent better parent-infant relationship. Internal consistency in the current sample was high for mothers (α = 0.89) and fathers (α = 0.85).

*Infant development* was assessed at T3 using the *communication* and *personal-social* subscales of the 14 months version of the Ages and Stages Questionnaire-3 (ASQ-3; Squires and Bricker, 2009), a brief parent rated questionnaire. Each domain contains six questions about age-specific developmental key-milestones (communication covers babbling, vocalizing, listening, and understanding; social-personal measures adaptive and social behaviors). Responses to each item is indicated as *yes* (0), *sometimes* (5), or *not yet* (10), depending on whether or not the infant is able to perform a certain task. For each scale, items are summed, resulting in total scale scores ranging from 0–60. In accordance with the questionnaire manual (Squires and Bricker, 2009), items were recoded so that higher scores represent better development. Internal consistency for the two ASQ-3 dimensions were somewhat low for mothers (communication α = 0.50, personal-social development α = 0.55) and fathers (communication α = 0.55; personal-social development α = 0.55).

*Confounders* pertaining to maternal, paternal, and infant characteristics assumed to be associated with the predictor and outcome were incorporated in the analyses. These included parental age and education [1 = *no school certificate*, 2 = *lower secondary education*, 3 = *secondary school certificate*, 4 = *advanced technical college entrance qualification*, 5 = *subject-related or higher education entrance qualification (A-level)*], both measured at T1. Moreover, infant characteristics such as infant gender (1 = *boys* and 2 = *girls*), prematurity, i.e., born before completion of gestational week 37 (0 = *not premature* and 1 = *premature*), and infant health (1 = *not healthy*, 2 = *healthy*) were assessed at T2. Further, parental postnatal depression was measured with the Edinburgh Postnatal Depression Scale (EPDS; Bergant et al., 1998) at T2. The scale ranges from 0–30. Higher scores indicate stronger symptoms of postnatal depression.

### Statistical Analyses

Descriptive data analyses and bivariate correlational analyses were conducted to acquire information on the present sample and about the relationships between the assessed variables. Dropout analyses were performed to examine potential differences regarding sociodemographic characteristics, predictors, and confounders between participants who did not complete T3 and those who did. Before running regression analyses, the main assumptions of the linear model were tested. Missing items on a psychometric scale were substituted with the person's mean score if <20% of the scale items were missing. Subsequently, multiple linear regression analyses were carried out to explore the prospective impact of maternal and paternal relationship satisfaction on infant developmental outcomes over time. To analyse mediating pathways through parent-infant relationship, the SPSS modeling tool PROCESS v3.5 macro by Hayes (2018) was used. Bootstrapping with 5,000 samples was applied to compute confidence intervals and inferential statistics. Effects are deemed as significant if the confidence interval does not include zero. If the effect of the predictor on the outcome is not statistically significant when entering the mediator to the model, a full mediation effect is assumed. If the effect remains significant but decreases in size, the mediator partially mediates the effect (Urban and Mayerl, 2018). Further, multiple regression analyses were used to compute standardized regression coefficients (β). To examine the possible moderating effects of infant gender, multiple regression analyses were conducted with mean centered predictors for a better interpretation of regression coefficients (Hayes, 2018). Potential confounders, i.e., parental age, education, depression, infant gender, infant health, and premature birth, were considered in the analyses. Forced entry was utilized, as recommended by Morgan and Winship (2014). To consider the possibility of differing maternal and paternal perspectives, analyses and accordingly all scale scores were calculated separately for mothers and fathers. Because of missing data and exclusion of extreme values, *n* varied between the different analyses. The analyses were conducted with statistical significance at *p* < 0.05 and performed using IBM SPSS Statistics 27.

### Ethical Statement

The DREAM study received ethical approval from the Ethics Committee of the Faculty of Medicine of the Technische Universität Dresden (No: EK 278062015). During recruitment, participants received written information about study aims and procedures. They were informed about pseudonymization and confidentiality as well as the possibility to withdraw from the study at any time. All participants gave written informed consent.

## Results

### Descriptive Statistics

The final sample consisted of *n* = 1,012 mothers and *n* = 676 fathers. Their characteristics are displayed in Table 1. Mean age of expectant mothers and fathers at T1 was 30.1 years (*SD* = 3.8) and 32.3 years (*SD* = 4.9), respectively. In the sample, 44.3% of expectant mothers and 47.7% of expectant fathers were married and 59.3% of expectant mothers and 58.3% of expectant fathers held a university degree. This indicates a rather high educational level of study participants compared to the overall German population (Statistisches Bundesamt, 2020). The majority of participants were expecting their first child (expectant mothers: 79.4%; expectant fathers: 78.9%). In both, the mothers' and fathers' subsamples, around half the infants were female (51.6% and 51.7%, respectively). Parents did not differ significantly in their assessments of parent-infant relationship and infant development (*p* > 0.05). Mothers were significantly more satisfied with their partnership, *t*_(1,617)_ = 3.30, *p* < 0.01 (mothers: *M* = 20.4, *SD* = 4.3; fathers: *M* = 19.7, *SD* = 4.0).

**Table 1:** Sample description.

	**Total (** ***n*** ^a^ **=** **1,688)**			
**Sample characteristics**	***n*** **(%** ^b^ **)**		***M*** **±** ***SD*** **(Range)**	
	**Mothers** **(*n* = 1,012)**	**Fathers** **(*n* = 676)**	**Mothers** **(*n* = 1,012)**	**Fathers** **(*n* = 676)**
Age in years^c^			30.1 ± 3.8 (19–43)	32.3 ± 4.9 (20–56)
Country of birth				
Germany	983 (97.1)	669 (99.0)		
Other	29 (2.9)	7 (1.0)		
Partnership status				
Married	447 (44.3)	322 (47.7)		
Unmarried	535 (53.0)	323 (47.9)		
Divorced	25 (2.5)	28 (4.2)		
Widowed	1 (0.1)	1 (0.1)		
Unknown	1 (0.1)	1 (0.1)		
Duration of the partnership in years			7.7 ± 4.0 (0–23)	7.8 ± 4.0 (0–20)
Parents living together				
Living permanently together	962 (96.2)	644 (96.6)		
Living together, but not permanently	35 (3.5)	20 (3.0)		
Living separated	3 (0.3)	3 (0.4)		
Parents living together (T3)^d^				
Permanently living together	988 (98.4)	662 (99.0)		
Not permanently living together	15 (1.5)	7 (1.0)		
Living separated	1 (0.1)	0 (0.0)		
Education				
No school certificate	1 (0.1)	0 (0.0)		
Lower secondary education	6 (0.6)	23 (3.4)		
Secondary school certificate	192 (19.0)	146 (21.8)		
Advanced technical college entrance qualification	85 (8.4)	53 (7.9)		
Subject-related or higher education entrance qualification (A-level)	728 (71.9)	449 (66.9)		
Professional education				
No university degree	411 (40.7)	278 (41.7)		
University degree	599 (59.3)	388 (58.3)		
Employment status^e^				
Full-time employed	461 (45.6)	557 (83.1)		
Part-time employed	171 (16.9)	56 (8.4)		
Others^f^	378 (37.4)	57 (8.5)		
Parity				
Nulliparous	796 (79.4)	519 (78.9)		
Primiparous	179 (17.8)	107 (16.3)		
Multiparous	28 (2.8)	32 (4.8)		
Relationship satisfaction (T2^g^; 0–27)^h^			20.4 ± 4.3 (4–27)	19.7 ± 4.0 (4–27)
Endearment			6.2 ± 2.1 (0–9)	5.8 ± 2.0 (0–9)
Community/communication			6.8 ± 1.7 (1–9)	6.9 ± 1.6 (2–9)
Disput behavior			7.4 ± 1.7 (0–9)	7.0 ± 1.7 (0–9)
Communicational development (T3; 0–60)^i^			45.3 ± 9.9 (20–60)	44.5 ± 10.6 (15–60)
Personal-social development (T3; 0–60)^i^			46.7 ± 10.7 (20–60)	46.0 ± 10.9 (20–60)
Parent-infant relationship (T2)^j^				
General factor (0–125)			112.2 ± 9.8 (32–125)	112.3 ± 8.4 (71–125)
Impaired bonding (0–60)			52.8 ± 5.7 (7–60)	53.1 ± 5.0 (27–60)
Rejection and anger (0–35)			32.3 ± 3.1 (9–35)	32.2 ± 2.7 (20–35)
Anxiety about care (0–20)			17.1 ± 2.0 (6–20)	16.9 ± 1.8 (8–20)
Risk of abuse (0–10)			10.0 ± 0.3 (5–10)	10.0 ± 0.1 (8–10)
Postnatal depression (T2; 0–25)^k^			5.6 ± 3.8 (0–25)	3.5 ± 3.2 (0–20)
Infant gender (T2)				
Female	516 (51.6)	341 (51.7)		
Male	484 (48.4)	318 (48.3)		
Infant age in weeks (T2)			8.5 ± 2.2 (3–23)	9.0 ± 2.2 (4–21)
Infant age in months (T3)			13.7 ± 0.5 (13–14)	13.8 ± 0.4 (13–14)
Infant health (T2)				
Healthy	979 (98.0)	650 (97.9)		
Ill	20 (2.0)	14 (2.1)		
Infant premature birth				
Premature birth	45 (4.5)	24 (3.6)		
No premature birth	966 (95.5)	652 (96.4)		

a *n slightly varies due to missing data of some participants;*

b *Valid percent;*

c *Unless stated otherwise, the data source is T1 = measurement point during pregnancy;*

d *T3 = 14 months after the actual birth date;*

e *Multiple answers possible;*

f *Including marginal employment, retraining, federal armed forces, apprenticeship, student, maternity leave, currently not working, and other;*

g *T2 = 8 weeks after the anticipated birth date;*

h *Short version of the Partnership Questionnaire (“Kurzform des Partnerschaftsfragebogens,” PFB-K);*

i *Subscale of Ages and Stages Questionnaire-3 (ASQ-3);*

j *Postpartum Bonding Questionnaire (PBQ) and it's subscales; reversed items so that higher scores indicate a higher level of parent-infant relationship*

k *Edinburgh Postnatal Depression Scale (EPDS)*.

### Dropout Analyses

Dropout analyses were conducted for all sociodemographic characteristics, predictors, and confounders of completers (T3 was completed) vs. non-completers (T3 was not completed), separately for mothers and fathers. In both subsamples, more completers than non-completers had a higher school-leaving qualification, mothers: *U* = 63,544.000, *Z* = −3.00, *p* < 0.01; fathers: *U* = 32,074.500, *Z* = −4.46*, p* < 0.01 and more completers held a university degree, mothers: *U* = 61,833.000, *Z* = −3.00 *p* < 0.01; fathers: *U* = 32,276.000, *Z* = −4.19, *p* < 0.01. In addition, female completers were less likely to have a premature birth than female non-completers (0.04% versus 0.09%), χ^2^(1) = 5.44, *p* = 0.02, and reported significantly lower levels of depression, *t*_(1,119)_ = 2.90, *p* < 0.01. In terms of parental relationship satisfaction, male completers were found to be significantly more satisfied than male non-completers, *t*_(766)_ = −2.27, *p* = 0.03. No further significant differences between completers and non-completers were observed.

### Direct Effect of Parental Relationship Satisfaction on Infant Development

Pearson correlations among all predictors, potential confounders, and outcomes were computed to explore their associations (mothers: see Table 2; fathers: see Table 3). In the maternal sample, there was no association between relationship satisfaction and the infant's personal-social (*r* = 0.05, *p* = 0.15) or communicational development (*r* = 0.02, *p* = 0.57). In the paternal sample, a positive correlation between relationship satisfaction and personal-social development (*r* = 0.11, *p* < 0.01) was revealed. Maternal (*r* = 0.11, *p* < 0.01) and paternal relationship satisfaction (*r* = 0.14, *p* < 0.01) were both significantly positively associated with parent-infant relationship. Parent-infant relationship showed a stronger association with infant development in fathers (communication: mothers: *r* = 0.08; *p* = 0.01, fathers: *r* = 0.12, *p* < 0.01; personal-social development: mothers: *r* = 0.08, *p* = 0.02; fathers: *r* = 0.13, *p* < 0.01).

**Table 2:** Pearson correlation coefficients (*r*) of predictors, control variables, and outcomes for mothers.

**Variable**	**1**	**2**	**3**	**4**	**5**	**6**	**7**	**8**	**9**	**10**
1. Communicational development	—									
2. Personal-social development	−0.47^**^	—								
3. Relationship satisfaction	−0.02	−0.05	—							
4. Mother-infant relationship	0.08^*^	0.08^*^	0.11^**^	—						
5. Parental age	−0.07^*^	−0.07	−0.07^*^	0.00	—					
6. Education	−0.03	−0.04	−0.01	−0.16	−0.06	—				
7. Depression	0.02	−0.02	−0.14^**^	−0.41^**^	−0.03	−0.03	—			
8. Infant gender	0.16^**^	0.18^**^	−0.04	0.00	−0.05	−0.03	0.02	—		
9. Infant health	0.03	0.01	0.06	−0.01	0.07^*^	0.04	0.05	0.01	—	
10. Premature birth	−0.09^**^	−0.14^**^	−0.03	0.03	−0.00	−0.04	−0.03	0.00	−0.07^*^	—

*Two-tailed-testing*.

* *p < 0.05*.

** *p < 0.01. N = 953*.

**Table 3:** Pearson correlation coefficients (*r*) of predictors, control variables, and outcomes for fathers.

**Variable**	**1**	**2**	**3**	**4**	**5**	**6**	**7**	**8**	**9**	**10**
1. Communicational development	—									
2. Personal-social development	0.47^**^	—								
3. Relationship satisfaction	0.01	−0.11^*^	—							
4. Father-infant relationship	0.12^**^	0.13^**^	0.14^**^	—						
5. Parental age	−0.02	−0.01	−0.02	0.05	—					
6. Education	−0.01	−0.03	−0.06	−0.07	−0.08	—				
7. Depression	−0.02	−0.08^*^	−0.23^**^	−0.39^**^	−0.07	−0.05	—			
8. Infant gender	0.15^**^	0.23^**^	−0.03	−0.00	−0.04	0.01	−0.03	—		
9. Infant health	0.01	0.03	0.01	0.00	0.04	0.06	0.06	0.04	—	
10. Premature birth	−0.06	−0.00	−0.05	0.01	−0.01	−0.02	−0.01	0.03	0.03	—

*Two-tailed-testing*.

* *p < 0.05*.

** *p < 0.01. N = 605*.

In order to examine the prospective impact of maternal and paternal relationship satisfaction on infant communicational and personal-social development over time, linear regression analyses were conducted. Parental age, education, depression, infant gender, infant health, and premature birth were included as confounders in all regression analyses. Paternal relationship satisfaction and infant personal-social development were positively associated (β = 0.08, *p* = 0.04; see Table 4), with *f* ^2^ = 0.09 indicating a small and medium effect (Cohen, 1988). No prospective association was found for mothers, nor was it found for either mothers or fathers with respect to communication development (see Supplementary Tables 1–3).

**Table 4:** Predictive value of paternal relationship satisfaction on infant personal-social development, controlled for parental age, education, depression, infant gender, infant health, and premature birth^a^.

**Modell**		***B***	***SE B***	**β**	**95% CI**	***p***
1	Paternal relationship satisfaction	−0.29	0.11	−0.11	[0.07; 0.52]	0.01
2	Paternal relationship satisfaction	−0.28	0.11	−0.10	[0.07; 0.50]	0.01
	Age	−0.00	0.10	−0.00	[−0.19; 0.18]	0.99
	Education	−0.69	0.46	−0.06	[−1.55; 0.25]	0.14
	Depression	−0.20	0.16	−0.05	[−0.52; 0.12]	0.23
	Infant gender	−5.19	0.87	−0.24	[3.51; 6.85]	0.00
	Infant health	−2.59	2.90	−0.04	[−8.45; 2.94]	0.35
	Premature birth	−0.95	1.45	−0.02	[−3.74; 1.92]	0.50

*Two-tailed-testing. B = unstandardized regression coefficient; SE B = standard error for unstandardized regression coefficient; β = standardized regression coefficient; CI = confidence interval; p = probability value. N = 585*.

a *Multiple linear regression, carried out by forced entry*.

For exploration, the significant association between paternal relationship satisfaction and infant personal-social development was investigated in more detail. To this end, it was examined whether the three subscales of the PFB-K (endearment, dispute behavior, and community/communication) are differently related to infant personal-social development. Endearment (β = 0.09; *p* = 0.03) and community/communication (β = 0.09; *p* = 0.02) were significantly associated with infant personal-social development. Dispute behavior (β = 0.05; *p* = 0.23) did not significantly relate to infant personal-social development (tables on request).

### Moderation Effect by Infant Gender

Moderation analysis was used to determine whether infant gender moderates the prospective association between paternal relationship satisfaction and infant personal-social development, when controlling for parental age, education, depression, infant gender, infant health, and premature birth. The analysis yielded a non-significant interaction term between paternal relationship satisfaction and infant gender (β = 0.01, *p* = 0.84), even when the individual subscales of the PFB-K were considered (endearment: β = 0.02, *p* = 0.70; dispute behavior: β = 0.00, *p* = 0.93; and community/communication: β = 0.00, *p* = 0.99). Thus, the prospective association between paternal relationship satisfaction and infant development was neither reduced nor increased as a function of infant gender.

### Mediation Effect by Father-Infant Relationship

**Figure 1:**
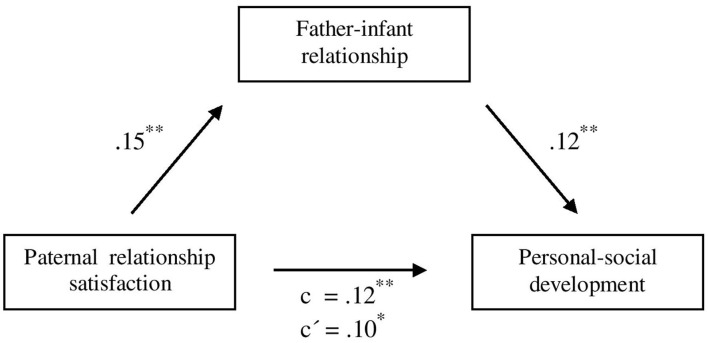
Mediation analysis for the prospective association of paternal relationship satisfaction on infant personal-social development, mediated by father-infant relationship, controlled for parental age, education, infant gender, infant health, and premature birth. Specification of the standardized regression coefficients (β); *c* = total effect; *c*′ = direct effect. *p < 0.05. **p < 0.01. *N* = 583–585.

To analyse whether the significant direct path between paternal relationship satisfaction and infant personal-social development could be explained by the father-infant relationship, mediation analysis was performed. As shown in Figure 1, higher values of paternal relationship satisfaction predicted a better father-infant relationship (β = 0.15, *p* < 0.01), which in turn predicted better personal-social development (β = 0.12, *p* < 0.01). The direct association between relationship satisfaction and personal-social development (β = 0.12, *p* < 0.01) was reduced when including father-infant relationship as a mediator (β = 0.10, *p* = 0.01). Thus, father-infant relationship partially mediated the relationship between paternal relationship satisfaction and infant personal-social development, when controlling for parental age, education, infant gender, infant health, and premature birth, indirect effect *ab* = 0.02, 95% CI [0.01; 0.09].

**Figure 2:**
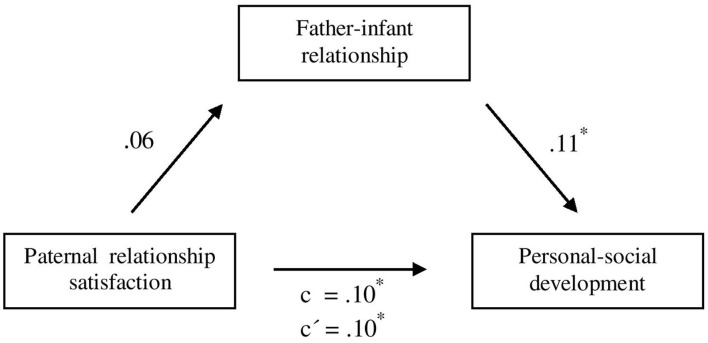
Mediation analysis for the prospective association of paternal relationship satisfaction on infant personal-social development, mediated by father-infant relationship, controlling for parental age, education, infant gender, infant health, premature birth and depression. Specification of the standardized regression coefficients (β); *c* = total effect; *c*′ = direct effect. *p < 0.05. **p < 0.01. *N* = 583–585.

When depression was included as another control variable, the mediation effect was no longer significant, indirect effect *ab* = 0.05, 95% CI [−0.00; 0.05]. As presented in Figure 2, the association between paternal relationship satisfaction and father-infant relationship, was no longer significant (β = 0.06, *p* = 0.10) when considering depression. Moreover, the association between father-infant relationship and personal-social development became slightly smaller (β = 11, *p* = 0.01). The direct associations between father-infant relationship and personal-social development (β = 11, *p* = 0.01) as well as between relationship satisfaction and personal-social development (β = 0.10, *p* = 0.01) remained stable when considering father-infant relationship (β = 0.10; *p* = 0.02).

An additional exploratory investigation was carried out to determine whether the mediation effect of the father-infant relationship differed for the various subscales of the PFB-K. The direct association between paternal relationship satisfaction and infant personal-social development was mediated by the father-infant relationship for all subscales when controlling for parental age, education, infant gender, infant health, and premature birth. When adding depression as a control variable the mediation effects were no longer significant (tables on request).

## Discussion

The present study sought out to explore the role of parental relationship satisfaction, a key aspect of family functioning (Garthus-Niegel et al., 2018), in infant development. Specifically, the aim was to investigate the prospective impact of maternal and paternal relationship satisfaction eight weeks postpartum on infant personal-social and communicational development one year later, controlling for parental age, education, depression, infant gender, infant health, and premature birth. Higher levels of maternal and paternal relationship satisfaction were hypothesized to be significant predictors of infant communicational and personal-social development. This prospective association was confirmed in the paternal sample with respect to infant personal-social development, whereas no such association was observed for mothers. Further, parental relationship satisfaction did not appear to be a significant predictor of infant communicational development. The strength of the prospective association between paternal relationship satisfaction and infant personal-social development did not vary as a function of infant gender. Findings further revealed that the father-infant relationship partly mediated the association between paternal relationship satisfaction and infant development. When controlling for paternal postnatal depression however, the mediation effect did not remain significant.

### Parental Relationship Satisfaction and Infant Development

Infant personal-social development was only significantly related to *paternal* relationship satisfaction. Accordingly, fathers who were more satisfied in their partnership reported better personal-social development of their infant when controlling for all potential confounders, and when the father-infant relationship was included. Detailed analyses of paternal relationship satisfaction revealed that infant personal-social development was mainly predicted by paternal perception of interparental endearment and affectionate community/communication. In contrast, fathers' assessment of arguing behavior yielded no prospective association with infant personal-social development. Although carried out in an exploratory manner, this finding suggests a great relevance of positive features of the couple relationship for optimal development in infancy. Similarly, Goldberg and Carlson (2014) showed that high interparental support is associated with less behavioral problems in children aged 3 to 9 years. Although, both mothers and fathers were included in their study, no distinction was made between them in the calculations. Therefore, no conclusions could be drawn about potential differences between mothers and fathers. In addition, Fishman and Meyers (2000) found that both maternal and paternal marital satisfaction were significantly associated with behavioral and emotional problems in children aged 5 to 9 years. Thus, in contrast to the results of the present study, an association between marital satisfaction and child outcomes was also found in the maternal sample. These differing results may be explained by the facts that children of different ages were included in the respective studies. Indeed, the prospective impact of parental relationship satisfaction on child outcomes may differ between developmental stages. To the best of our knowledge, no prior study included both parents when investigating how relationship satisfaction may predict development in infancy. Therefore, the current finding of a positive predictive association only in the paternal but not in the maternal sample highlights the need for inclusion of both, mothers and fathers in future research. However, it must be noted that the partner is of great importance for the level of paternal relationship satisfaction—as measured with the PFB-K. For instance, in the paternal PFB-K, the man is asked how often the woman hugs him or tells him that she loves him. Hence, the extent to which it is the mother's loving, affectionate behavior in the partnership that has a predictive impact on the infant's development warrants further research. The direct association between paternal relationship satisfaction and father-infant relationship could be explained by processes of social learning, in which the infant observes and imitates the parents' prosocial, affectionate interactions. For example, the infant may have learned to hug their dolls or soft toys (Item of the ASQ-3; Personal-social development) by watching their parents hug each other (Item of the PFB-K; Endearment).

Contrary to these findings, further analyses showed that in neither the mothers' nor the fathers' sample, infant communicational development was predicted by parental relationship satisfaction. This could be due to the fact that parental relationship satisfaction experienced in infancy may only have an effect on child communicational abilities in later life. Children's exposure to inter-parental interaction does often occur in coexistence with various other risk or protective factors (Dominick, 2018). The presence of multiple stressors or resources throughout childhood can confer decreased or increased risk and therefore explain the link between earlier experiences of parental relationship satisfaction and long-term effects on child communicational development. Thus, parental relationship satisfaction could have a delayed effect on child communicational development. Because research on maternal and paternal relationship satisfaction as a predictor of infant communicational development is rare, clarification of this long-term relation should be the subject of further investigations.

### Infant Gender

Furthermore, moderation analysis revealed no difference in how paternal relationship satisfaction was associated with boys' vs. girls' personal-social development. This absence of moderation by infant gender is consistent with previous research. For instance, Zhou et al. (2017) reported no child gender differences regarding the association between interparental conflict and behavior problems in two-year-old children. In addition, Goldberg and Carlson (2014) found that the association between interparental supportiveness and child internalizing as well as externalizing behavior problems in 3 to 9-year-old children was not moderated by child gender. Studies that report a difference between boys and girls mostly relate to older children. For them, cognitive factors such as different appraisals and self-accusations may be of explanatory importance, which, however, seem more relevant in children aged ~10 years and older (Dominick, 2018). In the first year of life, it appears that all infants, regardless of gender, benefit from parents' tenderness, support, and constructive communication with each other. Because of the previous ambiguous results and lack of prior investigations focussing on infancy, this research question was investigated in an exploratory way and therefore the results need to be confirmed in further analyses.

### Parent-Infant Relationship

The prospective association between paternal relationship satisfaction and infant personal-social development was partly explained by the father-infant relationship. In agreement with spillover theory, our results support the notion that feelings or behaviors generated in the interparental relationship may be transferred to the father-infant relationship. Accordingly, fathers who were more satisfied with their current partnership also had a better relationship with their infant, which in turn was associated with better infant personal-social development. The finding that paternal relationship satisfaction predicted father-infant relationship is in line with previous studies demonstrating that emotions, affect, and mood produced in the partnership carry over into parenting behaviors. Indeed, withdrawal from the family and hostility toward the infant were found to be common reactions to interparental disagreements especially among men (Harold et al., 2016; Zhou et al., 2017; Dominick, 2018). Moreover, a better father-infant relationship, in turn, significantly predicted increased infant personal-social development. Similarly, previous studies have shown that parent-child involvement significantly predicts children's emotional well-being (Fishman and Meyers, 2000) and that parenting behavior predicts child behavior (Feldkötter et al., 2019). However, when paternal postnatal depression was included as a control variable, the mediation effect disappeared. Fathers reporting higher levels of depression indicated lower relationship satisfaction, a poorer father-infant relationship, and poorer personal-social development of the infant. Hence, not surprisingly, the postnatal period represents a time of adjustment for fathers, including redefining the relationship and role distributions with their partner and learning to respond adaptively to their infant (Tsivos et al., 2015; Garthus-Niegel et al., 2020). Paternal relationship satisfaction did not explain the father-infant relationship beyond the contribution of depression, highlighting the importance of postnatal depression for the entire family. Previous studies have shown that partner support is essential for mental health, especially among fathers. In this regard, Garthus-Niegel et al. (2020) showed that perceived social support and relationship satisfaction serve as protective factors against paternal depression symptoms. Similarly, Røsand et al. (2012) reported that relationship satisfaction strongly buffers the effects of emotional distress in both men and women. Depressive symptoms in fathers at two months following birth in turn were found to predict a higher risk of behavioral problems in 3.5-year-old children (Ramchandani et al., 2005). According to these results, paternal depression may act as a mediator between paternal relationship satisfaction and infant personal-social development. This, as well as the role of the father-infant relationship, should be subject of further research. The major impact of depression is supported in numerous other studies indicating that parental depression has profound and widespread effects on interparental conflicts, parenting skills, parent-child relationship, and child development (e.g., Tsivos et al., 2015; Zhou et al., 2017; Dominick, 2018; Garthus-Niegel et al., 2018).

Although less strong than among fathers, positive associations were found between maternal relationship satisfaction and mother-infant relationship as well as between mother-infant relationship and infant development. Taken together, it seems that a higher level of parental relationship satisfaction is linked with a better parent-infant relationship, which in the case of fathers, is associated with personal-social infant development.

### Strengths and Limitations

Noteworthy strengths of this study are the longitudinal approach and the large sample size, comprising more than 1,600 parents. This study also includes both maternal and paternal perspectives, as well as the extended view of relationship quality, not focused on interparental conflicts and violence, but taking into account positive partnership features such as endearment and community/communication. Whereas most prior studies examined child development during late childhood and adolescence, this study focused on infancy, because these early months are a particularly formative time with an enormous impact on the infant's future life (Montada, 2008; Harold et al., 2016; Feldkötter et al., 2019).

Even though the study results extend the existing literature by providing important findings, they should be interpreted in light of several limitations. First, infant development was assessed through the parents' reports and therefore could be biased by parental perceptions. Although these reports provide an important perspective, it would be of interest to supplement this approach by means of independent observers and via standardized observational procedures in the future (e.g., using the Bayley Scales of Infant and Toddler Development; Bayley, 2006). Second, dyadic effects between partners were not taken into account. For this purpose, the Actor-Partner Interdependence Model (APIM; Kenny, 1996; Kashy and Kenny, 1999) offers a suitable framework because it maintains each individuals' unit measures but treats them as nested within the dyad. However, in the present study it was decided against a dyadic model in order to include those participants whose partner did not participate in the study and thus obtain a larger sample size. Third, even though the ASQ-3 is one of few recommended universal screening tools qualified to investigate children's development in infancy (Garthus-Niegel et al., 2017), internal consistency measured by Cronbach's α was rather low when assessing communicational and personal-social development. Low internal consistency tends to indicate low measurement accuracy and thus may lead to unreliable results. However, broad constructs such as the two developmental domains investigated in the current study are naturally expected to have lower item correlations than item collections reflecting a narrow, more tightly defined construct. Accordingly, lower reliability estimates can be expected for broad constructs (Garthus-Niegel et al., 2017). Fourth, it should be noted that parental relationship satisfaction and parent-infant relationship were assessed at the same time (T2). Due to this lack of temporal sequence between predictor and mediator, the mediation effect should not be interpreted in a causal sense. Lastly, participants were predominantly well-educated, first-time parents. Systematic dropout occurred in the maternal sample among mothers with a preterm birth and higher level of depression, whereas in the paternal sample, dropout occurred among fathers who were dissatisfied with their interparental relationship. Therefore, caution is warranted when generalizing the study findings to other populations. Nonetheless, selection bias may not necessarily influence the results when associations between variables are investigated (Nilsen et al., 2009).

### Implications

Future research should investigate whether current findings on the prospective association of relationship satisfaction and infant development can be replicated in other populations, e.g., populations with lower educational levels or more parents already having children. Additionally, it would be instructive to consider additional variables that may contribute to a more comprehensive understanding of this association. For example, the frequency of contact of the infant with others from whom it can learn and imitate communicative and personal-social behavior could be taken into account. Because the current analyses demonstrate the important role of paternal depression, future research should examine the extent to which the prospective association between paternal relationship satisfaction and infant personal-social development is mediated by paternal depression. Moreover, it would be of interest to further investigate the non-significant results regarding the three subscales of PFB-K (endearment, dispute behavior, and community/communication), in addition to the overall score.

In light of the study findings, it seems reasonable that (preventive) interventions for infant personal-social development should not only focus on the infant, but also comprehensively incorporate the couple relationship, the parent-infant relationship, and parental depression (Zemp and Bodenmann, 2015). Paternal relationship satisfaction could, for example, be strengthened by enhancing couples' interpersonal skills in the areas of open and benevolent communication and emotional empathy. Parent-infant relationships could for instance be strengthened through supporting paternal sensitivity and responsiveness to the infant's signals, as well as through parenting skills and strategies. As indicated by Tsivos et al. (2015), parent-infant therapy and a coaching intervention to promote parental responsiveness have the greatest efficacy for reducing symptoms of postnatal depression. Because the mother is of great importance for the father's relationship satisfaction and because the results suggest that both mother- and father-infant relationship are significantly related to infant development, both parents should be involved in these prevention or intervention approaches. Nevertheless, the findings of this study, namely the different impact of mothers and fathers on infant communicational and personal-social development, should be investigated further in order to develop more targeted measures for mothers and fathers.

## Conclusion

Taken together, the study findings emphasize the interconnectedness of family relations and individual development. The results indicate a direct prospective association between the father's relationship satisfaction and infant personal-social development. In addition, part of the association appears to be mediated by father-infant relationship. However, this mediation effect does not hold when postnatal depression is taken into account. Both girls and boys benefit from high parental relationship satisfaction. Future research should focus on this finding with consideration of additional factors that might mediate or moderate this prospective association. Hence, the current findings underscore the great relevance of paternal relationship satisfaction, father-infant relationship, and postnatal depression for infant personal-social development. Resulting from these findings and because the first years of life form the foundation for further development and long-term mental health (Harold et al., 2016; Feldkötter et al., 2019), prevention and intervention programmes that strengthen parental relationship satisfaction, parent-infant relationship, and postnatal depression are needed.

## Data Availability Statement

The datasets presented in this article are not readily available because of legal and ethical constraints. Public sharing of participant data was not included in the informed consent of the study. Requests to access the datasets should be directed to Susan Garthus-Niegel, susan.garthus-niegel@uniklinikum-dresden.de.

## Ethics Statement

The studies involving human participants were reviewed and approved by the Ethics Committee of the Faculty of Medicine of the Technische Universität Dresden (EK 278062015). The patients/participants provided their written informed consent to participate in this study.

## Author Contributions

CN performed the statistical analyses, drafted the initial manuscript, and reviewed and revised the manuscript. VK and MK supported the conduction of the study, especially through data collection, contributed with the interpretation of the data, and prepared the data for statistical analyses. SG-N acquired the funding, was responsible for conception and design of the basic DREAM study with its sub-studies as well as the coordination and supervision of the data collection and the ongoing cohort study, and contributed with the interpretation of the data. All authors contributed to manuscript revision, read, and approved the submitted version.

## Conflict of Interest

The authors declare that the research was conducted in the absence of any commercial or financial relationships that could be construed as a potential conflict of interest.

## Publisher's Note

All claims expressed in this article are solely those of the authors and do not necessarily represent those of their affiliated organizations, or those of the publisher, the editors and the reviewers. Any product that may be evaluated in this article, or claim that may be made by its manufacturer, is not guaranteed or endorsed by the publisher.
