# Supplementary material for: The Impact of Parental Relationship Satisfaction on Infant Development: Results From the Population-Based Cohort Study DREAM
Source: Front Psychol. 2021 Aug 6;12:667577. doi: 10.3389/fpsyg.2021.667577 (PMC8378853; doi:10.3389/fpsyg.2021.667577)
Supplement: Supplementary file 1 [file Data_Sheet_1.PDF]

Supplementary Material

Supplementary Figure 1. Study design of the DREAM study

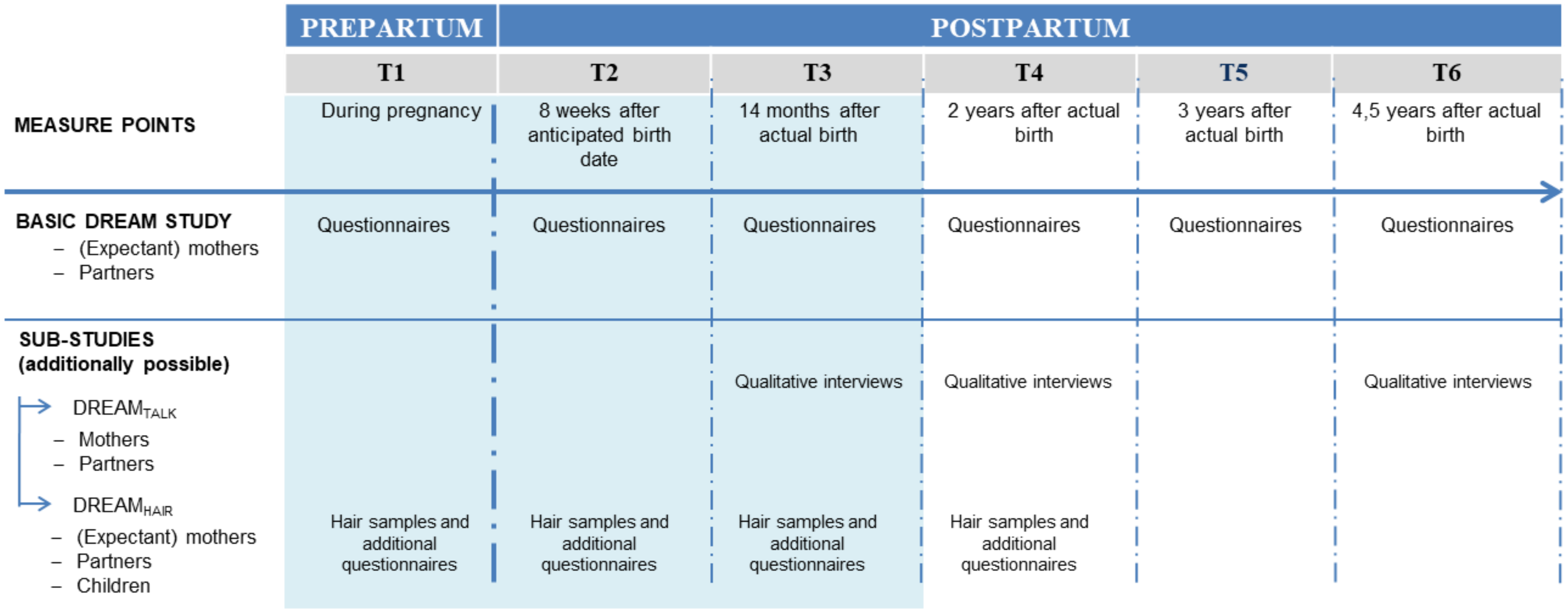

Supplementary Figure 2. Flowchart of retention rate and exclusion criteria resulting in final sample.

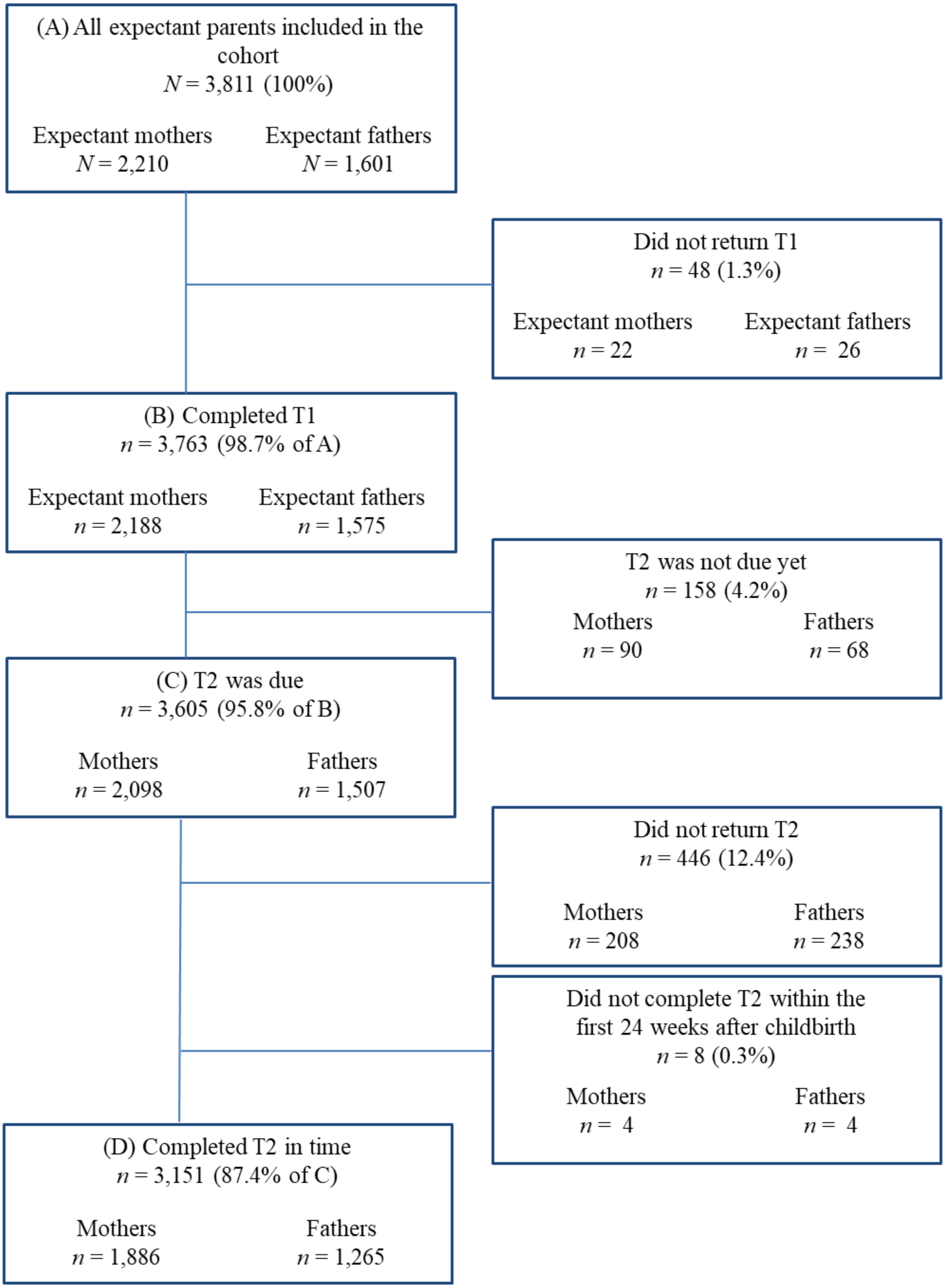

Figure 1. (continued)

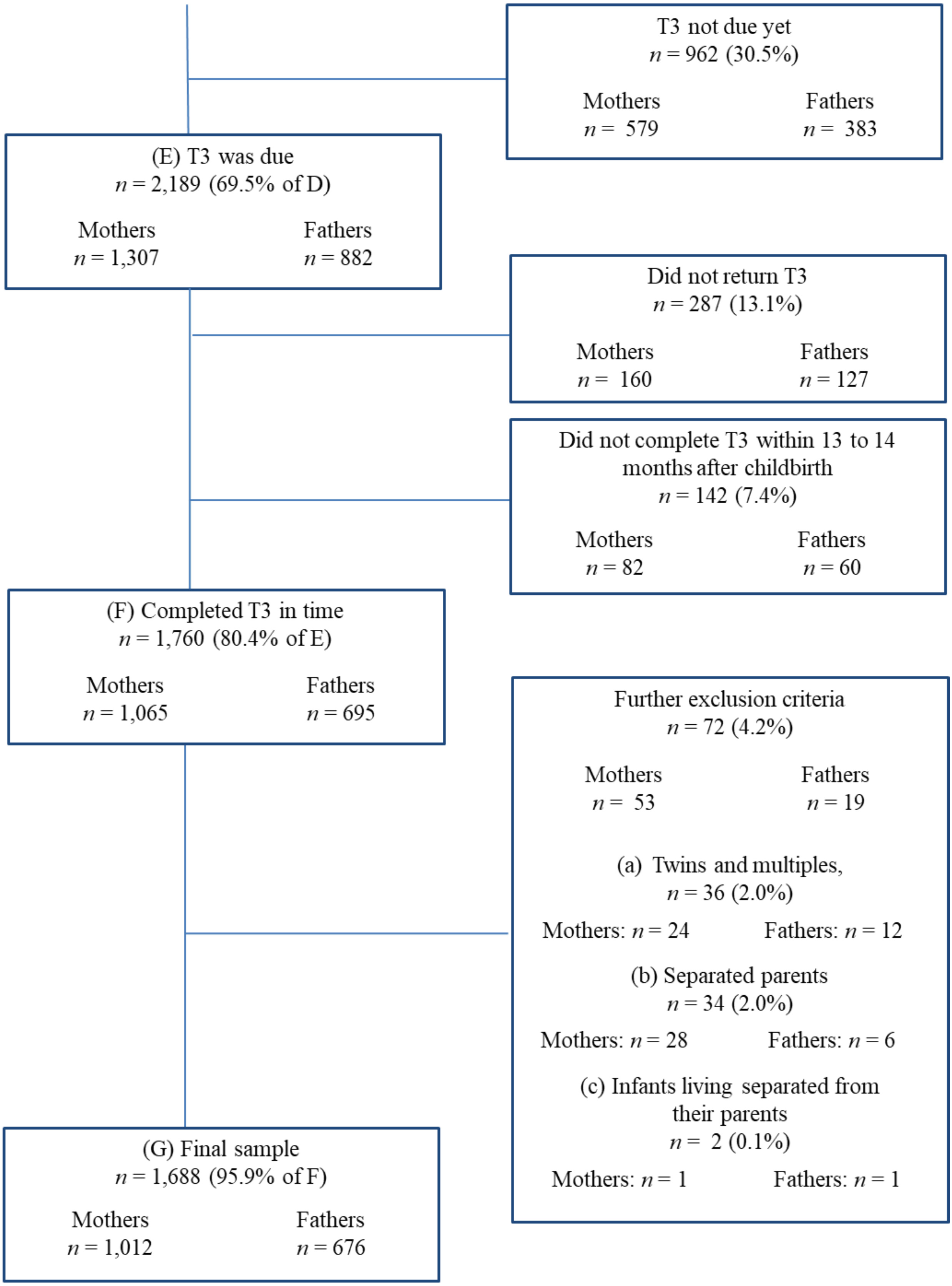

Note. T1 = during pregnancy; T2 = 8 weeks after the anticipated birth date; T3 = around 14 months after actual birth date. Data from 3rd of December 2020.

Supplementary Table 1. Predictive value of maternal relationship satisfaction on infant communicational development, controlled for parental age, education, depression, infant gender, infant health, and premature birth[a]

Modell		*B*	*SE B*	β	95% CI	*p*
1	Maternal relationship satisfaction	0.05	0.08	.02	[-0.09; 0.20]	.50
2	Maternal relationship satisfaction	0.06	0.08	.02	[-0.09; 0.21]	.47
	Age	-0.12	0.08	-.05	[-0.28; 0.04]	.16
	Education	-0.28	0.38	-.02	[-1.03; 0.49]	.46
	Depression	0.06	0.08	.02	[-0.10; 0.23]	.48
	Infant gender	3.04	0.64	.15	[1.72; 4.21]	.00
	Infant health	2.10	2.18	.03	[-2.21; 6.20]	.34
	Premature birth	-4.15	1.75	-.09	[-7.50; -0.66]	.02

Note. Two-tailed-testing. *B* = unstandardized regression coefficient; *SE B* = standard error for unstandardized regression coefficient; β = standardized regression coefficient; CI = confidence interval; *p* = probability value. *N* = 943.

[a]Multiple linear regression, carried out by forced entry.

Supplementary Table 2. Predictive value of maternal relationship satisfaction on infant personal-social development, controlled for parental age, education, depression, infant gender, infant health, and premature birth[a]

Modell		*B*	*SE B*	β	95% CI	*p*
1	Maternal relationship satisfaction	0.12	0.08	.05	[-0.03; 0.29]	.15
2	Maternal relationship satisfaction	0.12	0.08	.05	[-0.04; 0.28]	.17
	Age	-0.11	0.09	-.04	[-0.29; 0.07]	.26
	Education	0.11	0.42	.01	[-0.73; 0.94]	.81
	Depression	-0.05	0.09	-.02	[-0.23; 0.14]	.58
	Infant gender	3.76	0.68	.18	[2.39; 5.07]	.00
	Infant health	0.69	2.28	.01	[-3.53; 5.23]	.75
	Premature birth	-4.17	1.61	-.08	[-7.29; -0.95]	.02

Note. Two-tailed-testing. *B* = unstandardized regression coefficient; *SE B* = standard error for unstandardized regression coefficient; β = standardized regression coefficient; CI = confidence interval; *p* = probability value. *N* = 937.

[a]Multiple linear regression, carried out by forced entry.

Supplementary Table 3. Predictive value of paternal relationship satisfaction on infant communicational development, controlled for parental age, education, depression, infant gender, infant health, and premature birth[a]

Modell		*B*	*SE B*	β	95% CI	*p*
1	Paternal relationship satisfaction	0.00	0.11	.00	[-0.22; 0.21]	.99
2	Paternal relationship satisfaction	-0.01	0.11	-.00	[-0.23; 0.20]	.96
	Age	-0.05	0.10	-.02	[-0.24; 0.15]	.62
	Education	0.22	0.50	.02	[-0.75; 1.21]	.67
	Depression	-0.03	0.15	-.01	[-0.32; 0.27]	.85
	Infant gender	3.38	0.87	.16	[1.72; 5.12]	.00
	Infant health	0.99	2.95	.01	[-4.98; 6.60]	.72
	Premature birth	-4.19	2.27	-.07	[-8.62; 0.35]	.07

Note. Two-tailed-testing. *B* = unstandardized regression coefficient; *SE B* = standard error for unstandardized regression coefficient; β = standardized regression coefficient; CI = confidence interval; *p* = probability value. *N* = 605.

[a]Multiple linear regression, carried out by forced entry.
